# Genome-Wide Analysis of the Glutathione *S*-Transferase Gene Family in *Capsella rubella*: Identification, Expression, and Biochemical Functions

**DOI:** 10.3389/fpls.2016.01325

**Published:** 2016-08-31

**Authors:** Gang He, Chao-Nan Guan, Qiang-Xin Chen, Xiao-Jun Gou, Wei Liu, Qing-Yin Zeng, Ting Lan

**Affiliations:** ^1^Functional Genomics and Protein Evolution Group, State Key Laboratory of Systematic and Evolutionary Botany, Institute of Botany, Chinese Academy of SciencesBeijing, China; ^2^The Key Laboratory of Medicinal and Edible Plants Resources Development of Sichuan Education Commission, Chengdu UniversityChengdu, China; ^3^College of Biological Sciences and Biotechnology, Beijing Forestry UniversityBeijing, China

**Keywords:** gene family, gene duplication, genome, enzyme activity, functional divergence

## Abstract

Extensive subfunctionalization might explain why so many genes have been maintained after gene duplication, which provides the engine for gene family expansion. However, it is still a particular challenge to trace the evolutionary dynamics and features of functional divergences in a supergene family over the course of evolution. In this study, we identified 49 Glutathione *S*-transferase (GST) genes from the *Capsella rubella*, a close relative of *Arabidopsis thaliana* and a member of the mustard family. *Capsella* GSTs can be categorized into eight classes, with tau and phi GSTs being the most numerous. The expansion of the two classes mainly occurs through tandem gene duplication, which results in tandem-arrayed gene clusters on chromosomes. By integrating phylogenetic analysis, expression patterns, and biochemical functions of *Capsella* and *Arabidopsis* GSTs, functional divergence, both in gene expression and enzymatic properties, were clearly observed in paralogous gene pairs in *Capsella* (even the most recent duplicates), and orthologous GSTs in *Arabidopsis*/*Capsella*. This study provides functional evidence for the expansion and organization of a large gene family in closely related species.

## Introduction

Glutathione *S*-transferases (GSTs; EC 2.5.1.18) are multifunctional proteins encoded by a large gene family that is found in most organisms. As classical phase II detoxification enzymes, GSTs mainly catalyze the conjugation of reduced glutathione (GSH) with a wide variety of reactive electrophiles (Hayes et al., 2005). In plants, GST proteins are involved in several crucial physiological and developmental processes, including xenobiotic (e.g., herbicides) detoxification, signal transduction, isomerization, and protection against oxidative damages, UV radiation, and heavy metal toxins (Dixon et al., 2010; Cummins et al., 2011). Based on amino acid sequence similarity and gene organization, plant GSTs have been categorized into eight classes: phi, tau, theta, zeta, lambda, dehydroascorbate reductase (DHAR), tetrachlorohydroquinone dehalogenase (TCHQD) and the class containing the γ-subunit of the eukaryotic translation elongation factor 1B (EF1Bγ) (Oakley, 2005; Lan et al., 2009; Dixon and Edwards, 2010a). We recently identified two new GST classes (hemerythrin and iota) in non-vascular plants (Liu et al., 2013). Among the ten GST classes, phi, tau, lambda, and DHAR GSTs are considered unique to plants (Frova, 2006).

In plants, tau and phi class GSTs are the most numerous and play important roles in detoxification of xenobiotics (Frova, 2003). Overexpression of tau or phi GSTs in plants can increase tolerance to oxidation, herbicides, salinity, and chilling (Roxas et al., 1997; Cummins et al., 1999; Karavangeli et al., 2005; Benekos et al., 2010; Sharma et al., 2014). These proteins also participate in non-catalytic functions, e.g., binding/transport and signaling (Marrs, 1996; Lieberherr et al., 2003; Kitamura et al., 2004). Lambda and DHAR GSTs do not exhibit activity toward xenobiotics but are considered to be involved in redox and thiol transfer reactions (Dixon et al., 2002a; Dixon and Edwards, 2010b). DHAR GSTs have key functions not only in the ascorbate-GSH recycling reaction but also in stress resistance (Kwon et al., 2003; Chen and Gallie, 2006; Ushimaru et al., 2006). Recent studies demonstrated that some stress-inducible lambda GSTs could selectively bind flavonols and serve as antioxidants (Dixon and Edwards, 2010b; Dixon et al., 2011). The theta and zeta GSTs have counterparts in the mammalian system and function mainly as GSH-dependent peroxidases and isomerases (Thom et al., 2001; Basantani and Srivastava, 2007). GSTs in EF1Bγ class contain two domains: a typical GST domain and an EF1Bγ domain. The GST domain of EF1Bγ class GSTs functions as GSH peroxidases (Vickers et al., 2004).

*Capsella rubella* is from the same family as *Arabidopsis thaliana*. *C. rubella* is a model species widely used for studying natural variation in adaptive traits, such as flowering time (Guo et al., 2012). This species is also a good model for understanding the evolution of self-fertilization (Guo et al., 2009). In *Arabidopsis*, the haploid set consists of five chromosomes, whereas its close relative *C. rubella* has *n* = 8 chromosomes (Boivin et al., 2004). The progenitors of the lineage leading to *A. thaliana* and *C. rubella* diverged approximately 10 million years ago (Acarkan et al., 2000; Koch and Kiefer, 2005). The *C. rubella* genome has been completely sequenced (Slotte et al., 2013), thus facilitating the understanding of the evolutionary relationship between *C. rubella* and its relative *A. thaliana* from the gene family level. In this study, we performed genome-wide annotation of the GST gene family of *C. rubella*. Through phylogenetic analysis with expression and functional assays, we provided detailed characterization of the organization, gene expression pattern, and enzymatic properties of the GST members. Extensive functional divergence was observed among members within tandem-arrayed GST clusters and between paralogous gene pairs. Through comparative analyses of this family in *C. rubella* and *A. thaliana*, we examined the lineage-specific loss/gain events, and divergences in expression and substrate specificity in the orthologous GSTs. The genome-wide, multifaceted approach we employed provides new insights into the process of gene family evolution between closely related species.

## Materials and Methods

### Gene Identification and Nomenclature

To identify putative GST members in *C. rubella*, we performed TBLASTN searches with default algorithm parameters in the *Capsella* genome database, version 1.0^1^, using 55 GST protein sequences of *Arabidopsis* (Dixon and Edwards, 2010a), 81 of *populus* (Lan et al., 2009), and 575 of other plants, animals, fungi, and bacteria (Supplementary Table S5) as queries. These 575 full-length GSTs represent 36 GST sub-families defined by the NCBI Conserved Domain Database (CDD; Marchler-Bauer et al., 2011). All potential candidates identified were examined using the Pfam^2^ and CDD^3^ database to confirm the presence of typical GST N- and C-terminal domains in their protein structures. Preliminary classification of GST genes into subfamilies was performed using phylogenetic analysis. The proteins, which clustered with soluble cytosolic GSTs, have an ancient monophyletic origin (Dixon and Edwards, 2010a). They were used in subsequent analyses. Next, *Capsella* GSTs were amplified from genomic DNA and mRNA from mixed tissues of *C. rubella*, cloned into the pGEM-T Easy Vector (Promega), and sequenced in both directions to verify the gene sequences. The primers used for gene amplification are listed in Supplementary Table S2. Complete manual curation of the gene sequences and structures based on expressed sequence tag (EST) databases and experimental support was further performed to rectify incorrect start codon predictions, splicing errors, missed or extra exons, and incorrectly predicted pseudogenes. For genes that went undetected by PCR (5 out of 49 in this study), their gene structures were assumed to be identical to those of their closest phylogenetic relatives. This approach was adapted from other studies (Meyers et al., 2003).

The nomenclature for *Capsella* GSTs follows the system suggested by Dixon et al. (2002b) for plant GSTs. A univocal name was assigned to each *Capsella* GST gene consisting of two italic letters *Cr* denoting the source organism, the family name (e.g., *CrGSTU, CrGSTF, CrGSTT, CrGSTZ, CrGSTL, CrTCHQD, CrDHAR*, and *CrEF1B*γ corresponding to tau, phi, theta, zeta, lambda, TCHQD, DHAR, and EF1Bγ classes, respectively) and a progressive number for each gene (e.g., *CrGSTU1*).

### Phylogenetic Analyses

Full-length amino acid sequences were aligned using MUSCLE software^4^ and adjusted manually with BioEdit (Hall, 1999). Phylogenetic analysis was performed using the maximum-likelihood (ML) method in PHYML software (Guindon and Gascuel, 2003) with the Jones, Taylor, and Thornton (JTT) amino acid substitution model. GRX2 protein from *Escherichia coli* was chosen as an out-group during phylogenetic analysis of the *Capsella* GST family, as cytosolic GSTs are thought to be derived from the GRX2 (Holm et al., 2006). For phylogenetic analysis of each GST class, members of the sister class were used as an out-group. One-thousand bootstrap replicates were conducted to obtain confidence support.

### Expression of GST Genes in *Capsella* Tissues

The expression patterns of *Capsella* GST members during growth under normal conditions were examined by reverse transcription PCR (RT-PCR). Seeds of *C. rubella* were germinated on agar plates (Murashige and Skoog, 1962) and vernalized at 4°C for 4 days. Then, the seeds were grown in growth chambers under normal conditions (14 h light/10 h dark cycle) at a temperature of 25°C/22°C (day/night). Seedling plants were transplanted to soil for 2 weeks and harvested for RT-PCR analysis. We isolated total RNA from rossette leaves, roots, and hypocotyl tissues of each plant and dry seeds using an Aurum Total RNA kit (Bio-Rad Laboratories). Total RNA was treated with RNase-free DNase I (Promega) and reverse transcribed into cDNA using a TaKaRa RNA PCR kit (AMV), version 3.0. Forty-nine specific primer pairs were designed (Supplementary Table S3). The *actin* gene (Carubv10013961m.g) was used as an internal control. PCR conditions were optimized to consist of an initial denaturation step of 3 min at 95°C, followed by 35 cycles of 30 s at 94°C, 30 s at 60°C and 30 s at 72°C, with a final extension of 5 min at 72°C. PCR products from each sample were analyzed on 1% agarose gel and were validated by DNA sequencing. Independent biological triplicates were used in all of the RT-PCR analyses.

Gene expression profiles of the *Capsella* GSTs were compared with expression data from *Arabidopsis* ecotype *Columbia-0* (*Col-0*; Schmid et al., 2005) available through the *Arabidopsis* eFP browser at BAR (Winter et al., 2007). The eFP browser was set to the developmental map, with absolute expression values for gene expression. In this study, genes with values below 20 units were considered to be not expressed (Winter et al., 2007). The microarray data sets used in this study include leaves at rosette stage (ATGE_89_A, ATGE_89_B and ATGE_89_C), roots at rosette stage (ATGE_9_A, ATGE_9_B and ATGE_9_C), hypocotyls at seedling stage (ATGE_2_A, ATGE_2_B and ATGE_2_C), and dry seeds (RIKEN-NAKABAYASHI1A and RIKEN-NAKABAYASHI1B).

### Putative Promoter Sequence Analysis

Gene promoter sequences were extracted 1000 pb upstream of the transcriptional start site of each *Capsella* GST. Plant CARE database^5^ was used to find putative *cis*–elements among the promoter sequences. Divergence between upstream sequences of each paralogous gene pairs was measured by the GATA program (Nix and Eisen, 2005), with window size set as seven and lower cutoff score 12 bit.

### Expression and Purification of Recombinant *Capsella* GST Proteins

To investigate the enzymatic functions of *C. rubella* GST proteins, 24 tau, 11 phi, three DHAR, and three zeta GSTs were selected for protein expression analysis and purification. The primers used to construct the GST expression vectors are listed in Supplementary Table S4. The products were subcloned into pET-30a expression vectors (Novagen) to obtain a 6×His-tag at the N-terminus. The resulting plasmids, pET-30a/GSTs, were transformed into *E. coli* BL21 (DE3) and verified by sequencing. The transformed *E. coli* cells were cultured at 37°C and grown until the optical density (*A*_600_) reached 0.5. A final concentration of 0.1 mM isopropyl-β-D-thiogalactopyranoside was added to each culture, and the cultures were incubated at 37°C or 20°C overnight. The cells were harvested by centrifugation (10,000 *g*, 3 min, 4°C), resuspended in binding buffer (20 mM sodium phosphate, 0.5 M NaCl, and 20 mM imidazole, pH 7.4), and disrupted by cold sonication. The resulting homogenate was subjected to centrifugation (10,000 × *g*, 10 min, 4°C) and the supernatant was loaded onto a Ni Sepharose High Performance column (GE Healthcare Bio-Sciences) that had been pre-equilibrated with binding buffer. The GST proteins that bound to the Ni Sepharose High Performance column were eluted with elution buffer (20 mM sodium phosphate, 0.5 M NaCl, and 0.5 M imidazole, pH 7.4). The particulate material, a small portion of the supernatant and the purified proteins were analyzed by sodium dodecyl sulphate-polyacrylamide gel electrophoresis (SDS-PAGE) consisting of a 10% separating gel and a 5% stacking gel.

### Enzyme Assays of *Capsella* GST Proteins

The enzyme activity of plant GSTs was measured using the following six substrates: 1-chloro-2,4-dinitrobenzene (CDNB) and 4-nitrobenzyl chloride (NBC), as described by Habig et al. (1974); 7-chloro-4-nitrobenzo-2-oxa-1,3-diazole (NBD-Cl), as described by Ricci et al. (1994); and cumene hydroperoxide (Cum-OOH), dehydroascorbate (DHA), and diphenyl ethers (Fluorodifen), as described by Edwards and Dixon (2005). All assays were carried out at 25°C. Protein concentrations were determined by measuring the absorbance at 280 nm.

## Results

### Identification of the GST Genes from the *C. rubella* Genome

Forty-nine full-length genes encoding putative cytosolic GST proteins were identified in the *C. rubella* genome (Supplementary Table S1). Among these 49 genes, two genes (*CrGSTF3* and *CrGSTU9*) were considered to be putative pseudogenes because one contained a frame shift disrupting the coding region and the other contained a premature stop codon. After revising the frame shifts by deleting two nucleotides or removing the stop codon, these two full-length sequences were included in the phylogenetic and gene expression analyses. Based on a conserved domain analysis, these 49 GST candidates were divided into eight classes. The tau and phi GSTs were most numerous, with 25 and 12 copies, respectively. The DHAR and zeta classes each contained three members. Both the lambda and EF1Bγ classes were each represented by two members, and the theta and TCHQD classes only had one member each.

**FIGURE 1:**
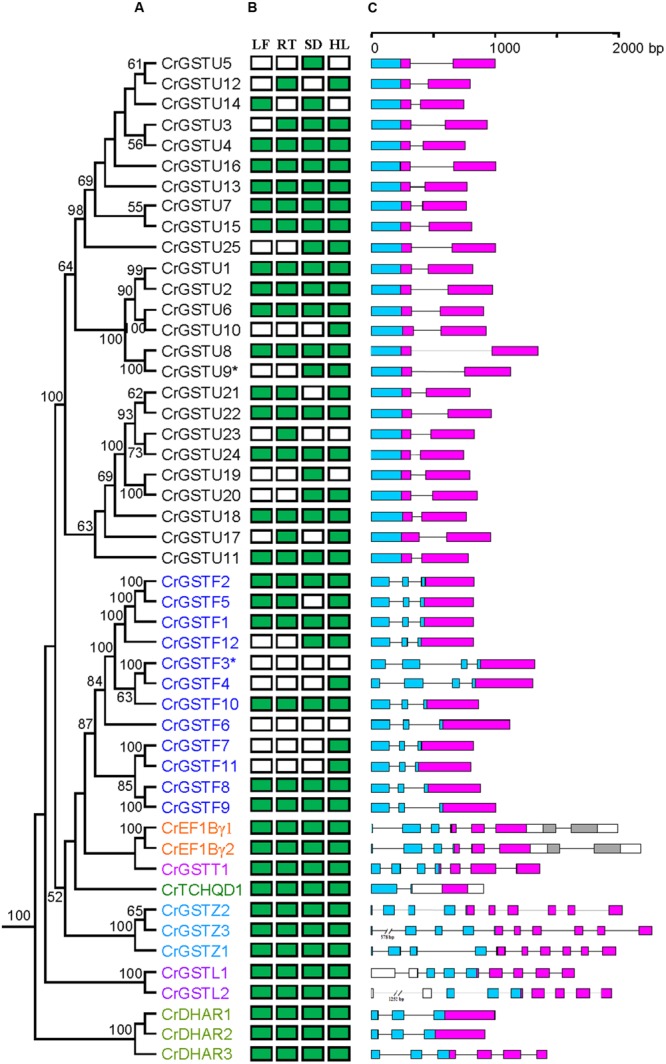
**Phylogenetic relationships among *Capsella* GSTs **(A)**, their expression patterns **(B)** and gene structures **(C)**.** Numbers at each node in the phylogenetic tree represent bootstrap values, and only values higher than 50% are shown. The Glutathione *S*-transferase (GST) genes belonging to different classes are indicated with different colors. In **(B)**, the green box indicates positive detection of gene expression in leaf (LF), root (RT), seed (SD), and hypocotyls (HL) under normal growth conditions. In **(C)**, the GST N- and C-terminal domain are highlighted by blue and purple boxes, respectively. Introns are shown as lines

Conserved gene structures were identified within each GST class. All 25 tau GST genes contained a one-intron/two-exon structure (**Figure 1C**). The intron positions were highly conserved among tau GST genes, and their lengths ranged from 70 to 642 bp. Ten of 12 phi GSTs had a two-intron/three-exon structure with a highly conserved first intron position. *CrGSTF3* and *CrGSTF4* contained a three-intron/four-exon structure. For the EF1Bγ, lambda, and zeta classes, the exon-intron architectures were conserved within each class, with each including 7, 9, and 10 exons, respectively.

### Genomic Organization of the *Capsella* GST Gene Family

**FIGURE 2:**
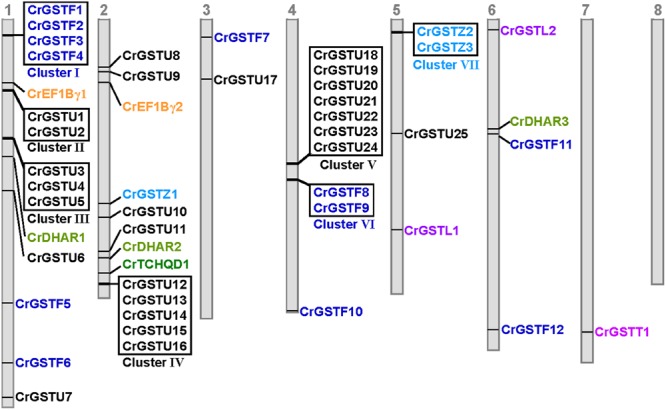
**Genomic localization of *Capsella* GSTs.** The numbered gray bars represent the eight *Capsella* chromosomes. The seven tandem-arrayed GST clusters are shown in boxes

The genomic locations of 49 full-length GSTs were assigned to all of the *Capsella* chromosomes except for chromosome 8 (**Figure 2**). The distribution of the GST genes among the chromosomes was obviously heterogeneous. Seven clusters (clusters I, II, III, IV, V, VI, and VII) with relatively high densities of GSTs were discovered on four chromosomes. In total, 51% of *Capsella* GST genes were organized in tandem repeats, indicating that tandem duplications significantly contributed to the expansion of the *Capsella* GST gene family.

Among the seven clusters, cluster V was the largest, consisting of seven tau GSTs in a 25-kb region on chromosome 4. These seven GST genes were oriented in the same direction. Cluster IV contained five tau GST genes that were clustered in a 10-kb region on chromosome 2. Three clusters (cluster I, II, and III) were located on chromosome 1. Cluster I harbored four phi GSTs arranged head-to-head in tandem in an 8-kb region. Clusters II and III contained two and three tau GSTs, respectively. Cluster VI contained two phi GST genes, and cluster VII contained two zeta GSTs.

### Phylogenetic Relationship of *Capsella* and *Arabidopsis* GST Gene Families

**FIGURE 3:**
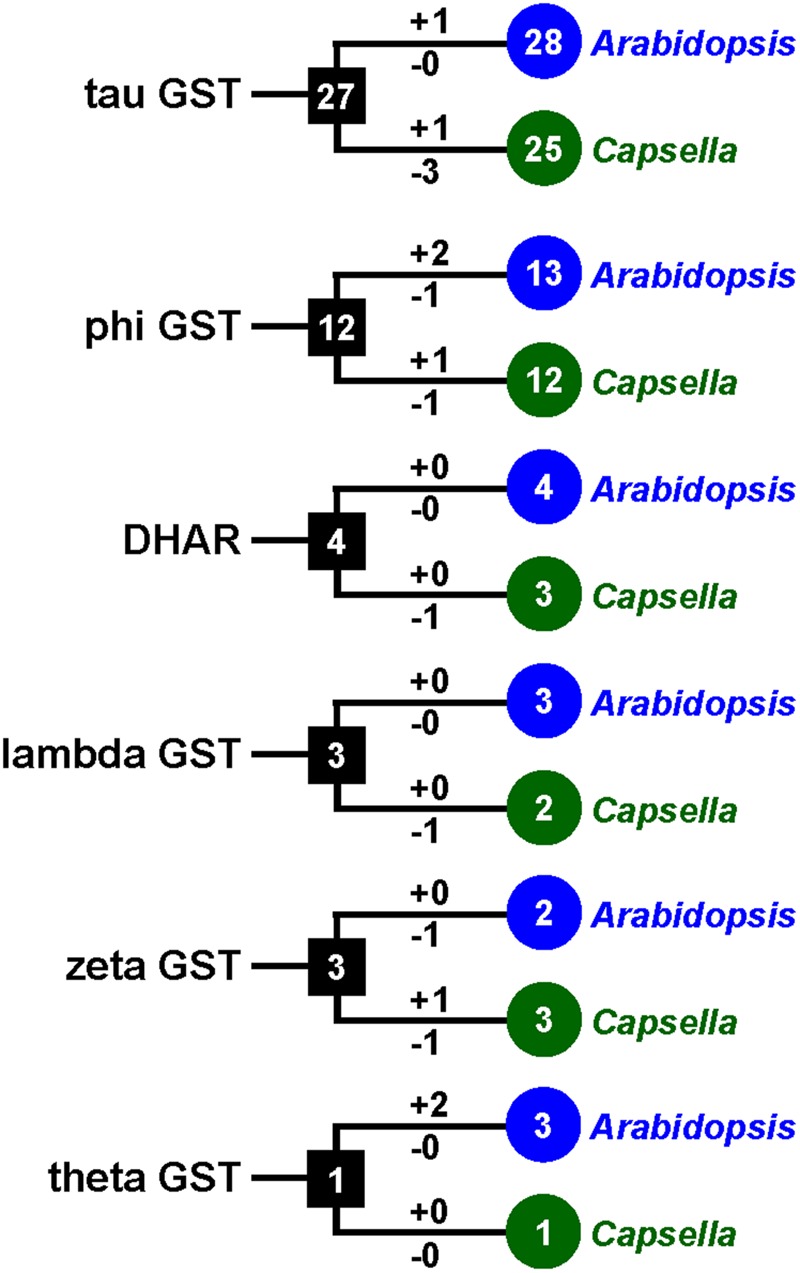
**The copy number changes of *Capsella* and *Arabidopsis* GSTs.** Numbers in circles and rectangles represent the numbers of GST genes in extant and ancestral species, respectively. Numbers on branches with plus and minus symbols represent the numbers of gene gains and losses, respectively.

**FIGURE 4:**
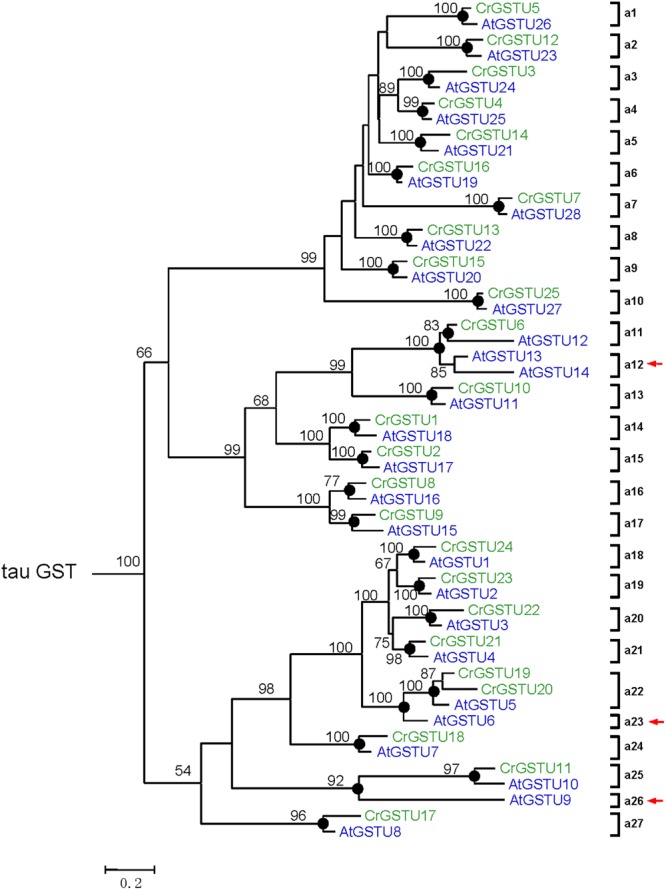
**Phylogenetic relationships of the *Capsella* and *Arabidopsis* tau GSTs.** Numbers at each node in the phylogenetic tree represent bootstrap values, and only values higher than 50% are shown. *Capsella* and *Arabidopsis* GSTs are indicated in green and blue, respectively. The nodes that represent the most recent common ancestral genes before the *Capsella* and *Arabidopsis* split are indicated by black circles. Clades that contain only *Capsella* or *Arabidopsis* GSTs are indicated by red arrows

To investigate the lineage-specific expansion of GST genes in the *Capsella* genome, we performed a phylogenetic analysis of all GSTs from *Capsella* and *Arabidopsis*. *Capsella* and *Arabidopsis* had 25 and 28 tau GST genes, respectively. There were at least 27 ancestral GST genes in the most recent common ancestor (MRCA) of *Capsella* and *Arabidopsis* (**Figures 3** and **4**). After the split, both *Capsella* and *Arabidopsis* gained one gene. However, *Capsella* had lost three genes, resulting in fewer tau GST genes in *Capsella* compared with *Arabidopsis*.

**FIGURE 5:**
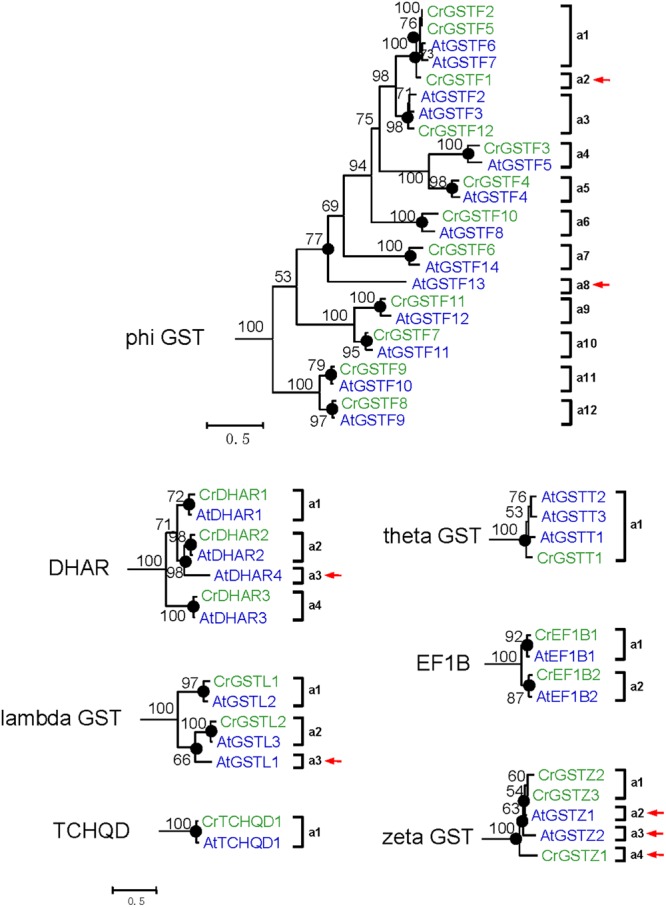
**Phylogenetic relationships of the *Capsella* and *Arabidopsis* phi and all six minor class GSTs.** Numbers at each node in the phylogenetic tree represent bootstrap values, and only values higher than 50% are shown. *Capsella* and *Arabidopsis* GSTs are indicated in green and blue, respectively. The nodes that represent the most recent common ancestral genes before the *Capsella* and *Arabidopsis* split are indicated by black circles. Clades that contain only *Capsella* or *Arabidopsis* GSTs are indicated by red arrows

*Capsella* and *Arabidopsis* had 12 and 13 phi GST genes, respectively. There were at least 12 ancestral GST genes in the MRCA of *Capsella* and *Arabidopsis* (**Figures 3** and **5**). After the split, *Capsella* and *Arabidopsis* gained one and two genes, respectively. Additionally, *Capsella* and *Arabidopsis* each lost one gene. Thus, *Capsella* contains one less phi GST gene than *Arabidopsis*.

For DHAR and lambda GSTs, there were at least four and three ancestral GST genes, respectively, in the MRCA of *Capsella* and *Arabidopsis* (**Figures 3** and **5**). After the split, *Capsella* lost a DHAR and lambda GST; however, the *Arabidopsis* genome did not lose any DHAR or lambda GST genes.

For zeta GST, the MRCA of *Capsella* and *Arabidopsis* had at least three ancestral zeta GSTs (**Figures 3** and **5**). After the split, the *Capsella* genome gained and lost one gene, and thus, *Capsella* still contains three zeta GSTs. *Arabidopsis* did not gain new copies. On the contrary, one zeta GST gene was lost, thus, the *Arabidopsis* genome contains two zeta GSTs.

For theta class GSTs, at least one ancestral GST gene was noted in the MRCA of the two species (**Figure 3**). Two duplication events in theta class GSTs were found in the *Arabidopsis* lineage after the split from *Capsella* (**Figure 5**). For TCHQD and EF1B class GSTs, we did not identify any gene gain or loss events after the split of these two species (**Figure 5**).

### Expression Patterns of the *Capsella* GST Gene Family

We examined the tissue-specific expression patterns of all 49 *Capsella* GSTs in four tissues, including leaves, roots, seeds, and hypocotyl zones using RT-PCR (**Figure 1B**). The expression patterns of the six minor class GSTs (*CrEF1B*γ*1* and *2, CrGSTT1, CrTCHQD1, CrGSTZ1, 2* and *3, CrGSTL1* and *2*, and *CrDHAR1, 2* and *3*) were homogenous, as these GSTs were expressed in all tissues examined in this study. Expression divergences were observed among the tau and phi class GSTs (**Figure 1B**). Of the 25 tau GSTs, 13 (*CrGSTU1, 2, 4, 6, 7, 8, 11, 13, 15, 16, 18, 22*, and *24*) were expressed in all tissues examined, and 12 genes (*CrGSTU3, 5, 9, 10, 12, 14, 17, 19, 20, 21, 23*, and *25*) were selectively expressed. *CrGSTU23* was exclusively expressed in root tissues. *CrGSTU5* and *CrGSTU19* were exclusively expressed in seed tissues, whereas *CrGSTU10* was only noted in hypocotyl zones. For the 12 phi GSTs, five (*CrGSTF1, 2, 8, 9*, and *10*) were expressed in all the tissues examined, and five (*CrGSTF4, 5, 7, 11*, and *12*) were selectively expressed. *CrGSTF3* and *CrGSTF6* were not expressed in any of the tissues examined (**Figure 1B**), which suggests that these two genes might exhibit loss of gene function by pseudogenization.

**FIGURE 6:**
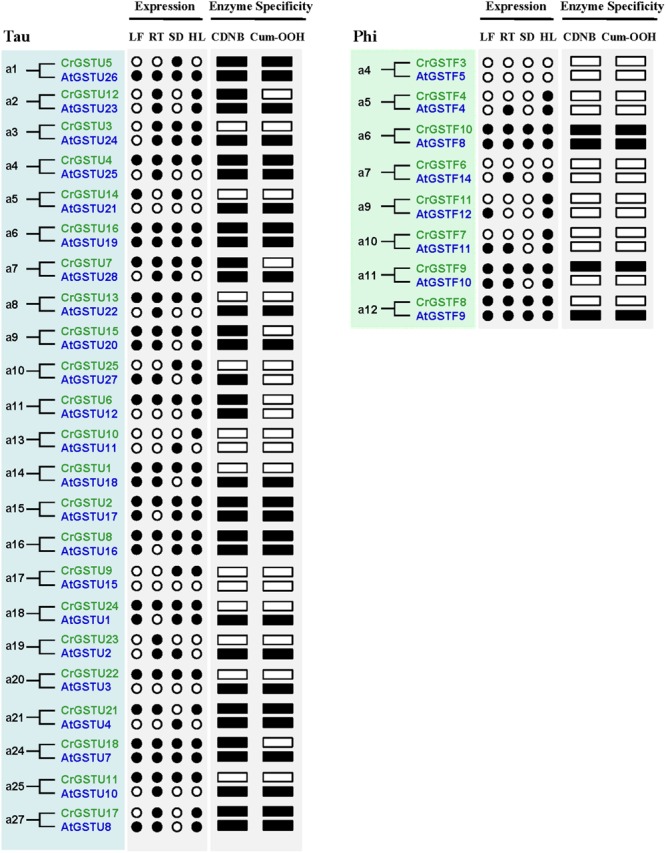
**Expression and functional divergence between ortholog gene pairs in *Capsella* and *Arabidopsis.*** The black circle and box indicate positive detection of gene expression in the corresponding tissue and specific activity toward 1-chloro-2,4-dinitrobenzene (CDNB) or cumene hydroperoxide (Cum-OOH), respectively.

The expression profiles of 23 tau and 8 phi orthologous GSTs in *Capsella* and *Arabidopsis* revealed a high degree of divergence (**Figure 6**). In total, 7 of the 31 orthologs displayed similar expression patterns, whereas the remaining 24 orthologs exhibited considerable expression divergence in some tissues. For example, *AtGSTU21* was not detected in any tissue of *Arabidopsis* (Goda et al., 2008; Kram et al., 2009), but its orthologous gene *CrGSTU14* was expressed in leaf and seed tissues (**Figures 1B** and **6**).

Potential regulatory motifs analysis using PlantCARE (Plant *cis*-acting regulatory element database) revealed a number of *cis*-elements in the promoter sequences of *Capsella* GST genes (Supplementary Table S6). These motifs were divided into at least eight functional categories, such as core promoters, ABA/abiotic stress, light, phytohormones, pathogen/elicitor/wound responsive elements as well as elements responsible for metabolism regulation, developmental stage, and organ specific expressions. The result showed considerable differences in the regulatory elements among the *Capsella* GSTs and within the subfamilies. Comparative analysis of upstream regions of close paralogs showed divergence, although there are conserved regions (Supplementary Figure S1), indicating that rapid divergence has occurred in the regulatory regions. Further experimental validation step is required to assess the changes in the *cis*-elements that are responsible for the expression diversity in GST genes.

### Substrate Specificities and Activities of *Capsella* GSTs]

In the *Capsella* genome, the tau and phi GSTs are most numerous, with 25 and 12 copies, respectively. The DHAR and zeta classes each contain three members. Thus, in this study, we selected tau, phi, DHAR, and zeta GSTs to express and purify GST proteins. Except for two pseudogenes (CrGSTU9 and CrGSTF3), Forty-one genes were cloned into expression vector pET-30a. Twenty-five of the 41 recombinant proteins were expressed as soluble proteins in *E. coli*, whereas the other 16 were insoluble. To determine the enzyme activity and substrate specificity of the soluble proteins, six substrates were selected: CDNB, NBD-Cl, NBC, Cum-OOH, and DHA.

**FIGURE 7:**
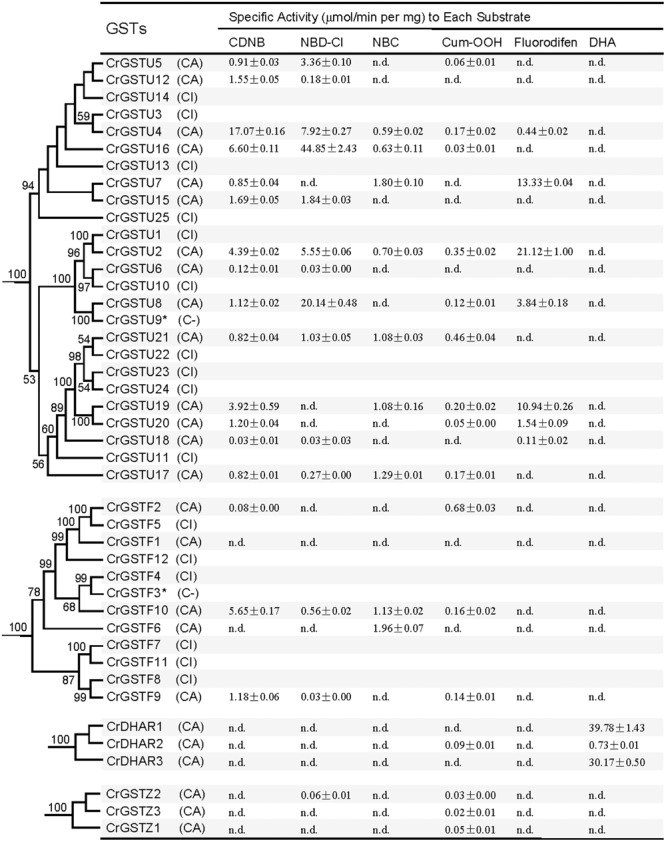
**Specific activities of the *Capsella* tau, phi, dehydroascorbate reductase (DHAR), and zeta class GSTs toward six substrates (Mean ± SD obtained from at least three independent determinations).** C, successfully cloned; A, purified GST protein assayed; I, recombinant protein totally insoluble; dash, analysis not performed; n.d., no activity detected.

For the tau GSTs, all 14 proteins showed specific activity toward CDNB, 11 toward NBD-Cl, nine toward Cum-OOH, and seven toward NBC and fluorodifen (**Figure 7**). Among the 14 tau GSTs, two (CrGSTU2 and 4) had enzymatic activity toward all five of the substrates. Five proteins exhibited activity toward four substrates, and four toward three substrates. Among the tau GSTs, CrGSTU4 showed the highest enzymatic activity toward CDNB, CrGSTU16 toward NBD-Cl, CrGSTU7 toward NBC, CrGSTU21 toward Cum-OOH, and CrGSTU2 toward fluorodifen. For the 12 phi GSTs, only five proteins were expressed as soluble proteins in *E. coli*. Among these five proteins, CrGSTF10 exhibited boarder substrate spectra and enzymatic activities for four substrates. CrGSTF1 did not exhibit any activity toward any of the tested substrates. All three DHAR GSTs exhibited activity toward DHA. Compared with CrDHAR1 and 3, CrDHAR2 had noticeably reduced enzymatic activity toward DHA, whereas CrDHAR2 exhibited activity toward Cum-OOH.

Substantial variations in specific activities toward different substrates were noted among the members of tandem-arrayed GST clusters. For example, CrGSTU12, 15, and 16 belong to cluster IV. CrGSTU16 displayed a much broader substrate spectrum than did CrGSTU12 and 15. Although CrGSTU12 and 15 shared a similar substrate spectrum, their specific activities toward NBD-Cl varied 10-fold (**Figure 7**).

The enzymatic activities of orthologous GSTs in *Capsella* and *Arabidopsis* also displayed variations. We made a comparison of enzyme specificity toward CDNB and Cum-OOH between orthologous GSTs (Dixon et al., 2009). For example, CrGSTU7, CrGSTU12, CrGSTU15, and CrGSTU18 had enzymatic activity for CDNB but no activity for Cum-OOH, whereas their orthologs in *Arabidopsis* had enzymatic activity for both substrates (**Figures 6** and **7**).

## Discussion

Functional divergence of duplicated genes is a major factor promoting their retention in the genome (Ohno, 1970; Zhang, 2003). Many theoretical models have been proposed to explain the mechanisms leading to the divergence include sub-functionalization, neo-functionalization, and dosage-balance model, etc (Ohno, 1970; Hughes, 1994; Force et al., 1999; Walsh, 2003; Moore and Purugganan, 2005; Veitia et al., 2008; Innan and Kondrashov, 2010). However, our understanding of the mechanisms driving the evolution of a large and functionally heterogeneous gene family is limited. Because to determine whether the duplicates have identical, similar, or different functions requires comprehensive examination of the functions of each gene product, while this approach is useful at a single gene level, genome-scale analyses of functional divergence of a supergene family are unfeasible. Our study combined bioinformatics and experimental approaches to explore the functional diversification of GST gene family at different levels of genomic organization: among subfamily classes, within tandem clusters, in paralogous and orthologous gene pairs.

Genome annotation identified 49 full-length GST genes from the *C. rubella* genome, which were divided into eight classes. Extensive study has showed that tau and phi classes were the most abundant with wide interspecific variation in copy number in plants (Lan et al., 2009, 2013; Dixon and Edwards, 2010a; Jain et al., 2010; Liu et al., 2013, 2015). For instance, our study and previous studies showed that tau GSTs was not found in moss and green algae, whereas it’s ubiquitous in tracheophytes (25–62 copies). Seventeen phi GSTs were found in rice while only two were represented in *S. moellendorffii*. However, other six classes remain comparatively small, with only 1–5 members. Comparison of copy numbers among the classes indicated that they might follow distinct evolutionary paths. Why did extensive expansion of tau and phi classes occur? A possible explanation is functional requirement. Tau and phi GSTs play an important role in the detoxification of xenobiotics and defense responses against both biotic and abiotic stresses (Loyall et al., 2000; Karavangeli et al., 2005; Benekos et al., 2010; Dixon et al., 2011; Jha et al., 2011; Cummins et al., 2013). Thus, the large scale expansion within tau and phi classes might provide defense against a broader range of xenobiotics and facilitated their tolerance to various environmental hazards. Our study exhibited extensive diversification in enzyme substrate specificity and transcript expression in tau and phi classes. This might further support the diversification in response to a set of changing substrates and regulatory properties.

The rapid expansion of GST gene family in plants is largely the result of the expansion of tau and phi classes. In the *C. rubella* genome, 17 of the 25 (68%) tau GSTs consisted of four clusters, and six of the 12 (50%) phi GSTs consisted of two clusters, indicating that tandem duplication considerably contributed to the expansion of tau and phi GSTs in the *C. rubella* genome. Previous studies also indicate that tandem duplication played important roles in the expansion of tau and phi GSTs in poplar, soybean, *Arabidopsis*, and rice genomes (Dixon et al., 2002b; Soranzo et al., 2004; Lan et al., 2009; Liu et al., 2015). Why have so many duplicate genes been retained for such a long time in the *C. rubella* genome? To investigate this question, we examined the seven tandem-arrayed clusters (Cluster I–VII). We found two categories of expression patterns. In the first, all the members in each cluster were expressed in all tissues. This pattern was observed in tau cluster II (*CrGSTU1*/*2*), phi cluster VI (*CrGSTF8*/*9*), and zeta cluster VII (*CrGSTZ2*/*3*; **Figure 1**). In the second category, found in tau cluster III (*CrGSTU3*-*5*), IV (*CrGSTU12*-*16*), V (*CrGSTU18*-*24*), and phi cluster I (*CrGSTF1*-*4*), some copies were expressed in all tissues, some had restricted tissue-specific expression or were not expressed in any tissue examined (**Figure 1B**). When enzyme assays were examined, no GST proteins in clusters showed identical enzymatic activities and specificities toward different substrates (**Figure 7**). Through this integrated approach, we found that rapid divergence has occurred in the regulatory regions of genes and in their biochemical properties within clusters, suggesting that partial sub-functionalization has indeed taken place. This seems to be an important factor promoting the duplicated GSTs’ retention in the genome.

A major challenge in comparative genomics is to find sufficient functional differences between species. However, it remains challenging in *Arabidopsis* and other plants, partly due to technical limits and potential functional redundancy within the family (Sappl et al., 2009). We identified 23 tau and 8 phi orthologous GSTs in the two relatives, and most of the gene pairs exhibited variations at expression and biochemical level (**Figure 6**), indicating that their functions may have evolved after the split. For example, *AtGSTF12* showed high expression in senescent leaf and was demonstrated to be involved in flavonoid metabolism (Kitamura et al., 2004; Dixon and Edwards, 2010a). But its orthologous gene *CrGSTF11* was not detected in leaf tissue (**Figure 1B**). AtGSTU25 and AtGSTU28 have the highest activity in tau class when assayed with CDNB or Cum-OOH as substrates (Dixon et al., 2009), whereas their orthologs CrGSTU4 and CrGSTU7 have low activity for Cum-OOH and CDNB, respectively (**Figure 7**). *AtGSTU20* was showed to interact with *Far-red (FR) insensitive 219* (*FIN219*) in response to light and could regulate cell elongation and plant development (Chen et al., 2007). We detected some light responsive elements in the promoter of *CrGSTU15* (Supplementary Table S6), suggesting that *CrGSTU15* may also involve in light regulation. In addition, 5 of the 31 orthologs displayed similar patterns (**Figure 6**). *AtGSTU19* and *CrGSTU16* displayed similar expression and substrate spectrum. *AtGSTU19* showed tolerance to salt, drought, and methyl viologen stresses (Xu et al., 2015). Several *cis*-acting elements involved in abscisic acid, anaerobic, heat, low-temperature, drought, defense, and phytohormones responsiveness were indentified in *CrGSTU16* as well, suggesting that this gene may also be induced by several stimuli. However, *AtGSTF8* and *CrGSTF10* exhibited a different example: As an enzyme, *AtGSTF8* was the most active member in phi class when assayed with CDNB and Cum-OOH (Dixon et al., 2009). Its expression was strongly induced by salicylic acid and H_2_O_2_ in root tissue, and *ocs* element in the promoter region played an important role (Chen and Singh, 1999). Unlike *AtGSTF8, CrGSTF10* didn’t contain *ocs*-like element and its specific activity toward Cum-OOH was low (Supplementary Table S6 and **Figure 7**). Protein subcellular relocalization is also considered as another form of functional divergence (Marques et al., 2008; Qian and Zhang, 2009; Wang et al., 2009). *AtGSTU12* is the only tau class GST localized entirely to the nucleus, containing a putative nuclear localization signal KKRKK (Takahashi et al., 1995). But we did not find this signal in *CrGSTU6*. All these results suggested that functional divergence previously had occurred in the two lineages after the split.

## Conclusion

In this study, we characterized the complete set of GST gene family in *C. rubella* genome. By phylogenetic and functional analysis, we compared it to that in *Arabidopsis*. We examined the gene gain and loss events after the divergence of the relatives. Also, we evaluated the functional divergences of recently expanded GSTs and orthologs. Through these analyses, we were able to draw a picture illustrating how gene duplication and sub-functionalization influence the divergence, retention, and functions of GST genes in the *Capsella* genome. Furthermore, by extending genome-wide comparison analysis of GST gene family with more species in the Brassicaceae, the study will provide a comprehensive overview of the evolutionary history of a large gene family among lineages and mechanism of functional diversification and retention of duplicates.

## Author Contributions

TL and QZ designed the study. GH, CG, and QC performed the experiments. TL, XG, and WL analyzed the data. TL wrote the paper.

## Conflict of Interest Statement

The authors declare that the research was conducted in the absence of any commercial or financial relationships that could be construed as a potential conflict of interest.

## Footnotes

1 https://phytozome.jgi.doe.gov/pz/portal.html

2 http://pfam.xfam.org/search

3 http://www.ncbi.nlm.nih.gov/Structure/cdd/wrpsb.cgi

4 http://www.drive5.com/muscle/

5 http://bioinformatics.psb.ugent.be/webtools/plantcare/html/
