# Supplementary material for: Genome-Wide Analysis of the Glutathione S-Transferase Gene Family in Capsella rubella: Identification, Expression, and Biochemical Functions
Source: Front Plant Sci. 2016 Aug 31;7:1325. doi: 10.3389/fpls.2016.01325 (PMC5005422; doi:10.3389/fpls.2016.01325)
Supplement: Supplementary file 1 [file Data_Sheet_1.PDF]

Supplementary Material

Genome-wide analysis of the glutathione S-transferase gene family in *Capsella rubella*: identification, expression and biochemical functions

Gang He[1,2,4], Chao-Nan Guan[3,4], Qiang-Xin Chen[1], Xiao-Jun Gou[2], Qing-Yin Zeng[1] and Ting Lan[1,*]

[1] State Key Laboratory of Systematic and Evolutionary Botany, Institute of Botany, Chinese Academy of Sciences, Beijing, China.

[2] The Key Laboratory of Medicinal and Edible Plants Resources Development of Sichuan Education Commission, Chengdu University, Chengdu, China

[3] College of Biological Sciences and Biotechnology, Beijing Forestry University, Beijing, China

[4] These authors contributed equally to the article.

[*]Corresponding author: Ting Lan

State Key Laboratory of Systematic and Evolutionary Botany

Institute of Botany, Chinese Academy of Sciences

Beijing, 100093, China

E-mail: lanting@ibcas.ac.cn

Phone: +86-10-62836491; Fax: +86-10-62590843

Running title: *Capsella* GST gene family

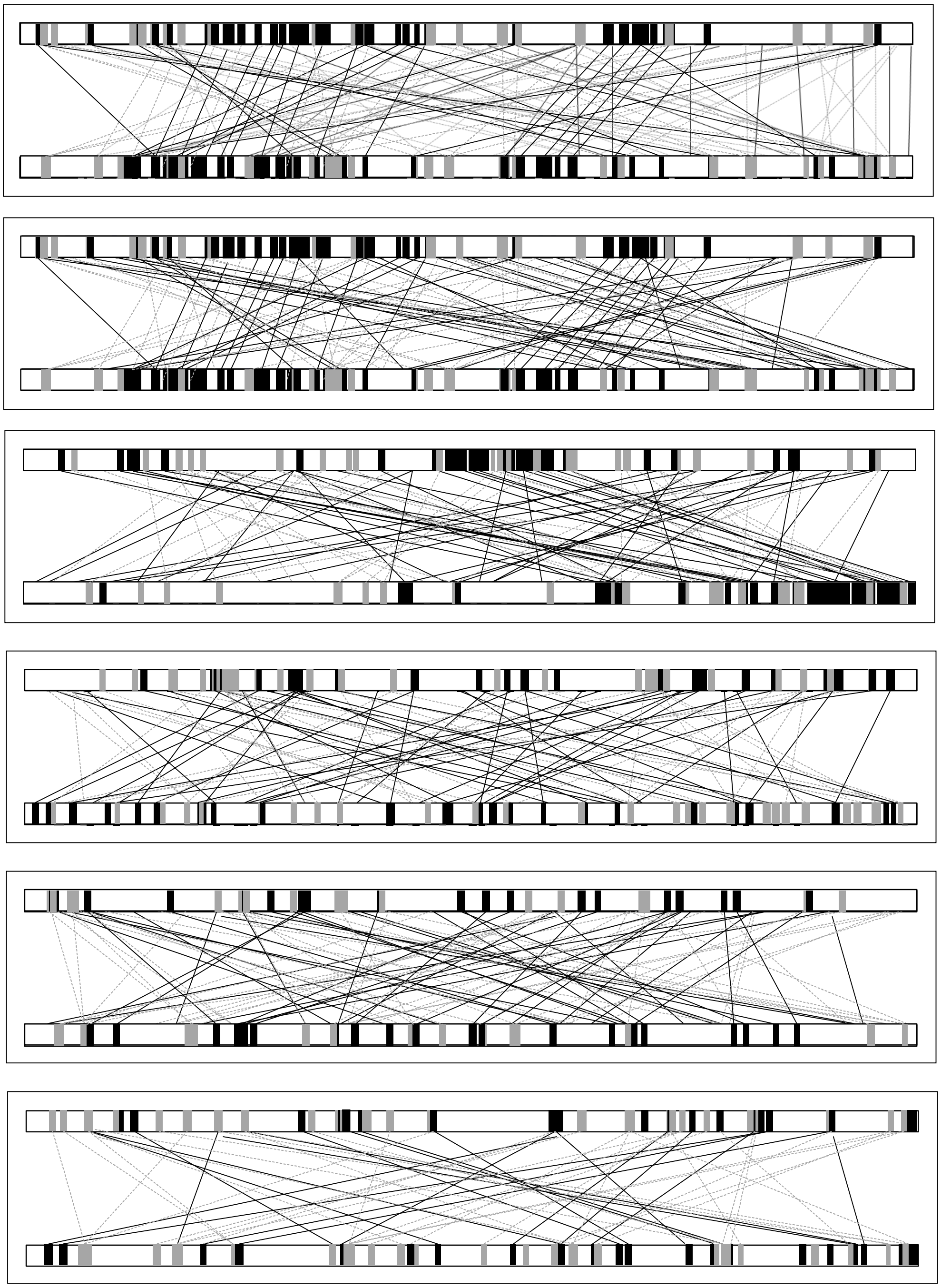

CrGSTF2
CrGSTF5
K_s = 0

CrGSTU19
CrGSTU20
K_s = 0.11

CrGSTZ2
CrGSTZ3
K_s = 0.18

CrGSTF3
CrGSTF4
K_s = 0.62

CrGSTU1
CrGSTU2
K_s = 0.76

CrGSTF8
CrGSTF9
K_s = 1.03

Supplementary Fig. S1 Comparative analysis of the 1000 bp upstream of paralogous gene pairs. Solid dark lines connect similar regions and gray broken lines connect matched regions in reversed orientation.

Supplementary Table S1 Full-length GSTs genes identified from the *Capsella rubella* genome. Putative pseudogenes are marked with an asterisk

Class	Gene Name	Accession number	Map position (bp)
Tau	*CrGSTU1*	KT935196	scaffold_1: 3480495 - 3481315
	CrGSTU2	KT935197	scaffold_1: 3482279 - 3483256
	CrGSTU3	KT935198	scaffold_1: 5927670 - 5928608
	CrGSTU4	KT935199	scaffold_1: 5930298 - 5931053
	CrGSTU5	KT935200	scaffold_1: 5932144 - 5933140
	CrGSTU6	KT935201	scaffold_1: 8546587 - 8547495
	CrGSTU7	KT935202	scaffold_1: 18884278 - 18885044
	CrGSTU8	KT935203	scaffold_2: 2434455 - 2435801
	*CrGSTU9**	KT935204	scaffold_2: 2457049 - 2458175
	CrGSTU10	KT935205	scaffold_2: 9786466 - 9787390
	CrGSTU11	KT935206	scaffold_2: 11607842 - 11608623
	CrGSTU12	KT935207	scaffold_2: 13155111 - 13155908
	CrGSTU13	KT935208	scaffold_2: 13156412 - 13157181
	CrGSTU14	KT935209	scaffold_2: 13161102 - 13161844
	CrGSTU15	KT935210	scaffold_2: 13162700 - 13163510
	CrGSTU16	KT935211	scaffold_2: 13164665 - 13165670
	CrGSTU17	KT935212	scaffold_3: 2883787 - 2884747
	CrGSTU18	KT935213	scaffold_4: 7218580 - 7219342
	CrGSTU19	KT935214	scaffold_4: 7223334 - 7224128
	CrGSTU20	KT935215	scaffold_4: 7231549 - 7232405
	CrGSTU21	KT935216	scaffold_4: 7234755 - 7235552
	CrGSTU22	KT935217	scaffold_4: 7240385 - 7241353
	CrGSTU23	KT935218	scaffold_4: 7241895 - 7242726
	CrGSTU24	KT935219	scaffold_4: 7243373 - 7244120
	CrGSTU25	KT935220	scaffold_5: 5730699 – 5731699
Phi	*CrGSTF1*	KT935181	scaffold_1: 699841 - 700668
	CrGSTF2	KT935182	scaffold_1: 702570 - 703399
	*CrGSTF3**	KT935183	scaffold_1: 704308 - 705626
	CrGSTF4	KT935184	scaffold_1: 706443 - 707748
	CrGSTF5	KT935185	scaffold_1: 14227526 - 14228352
	CrGSTF6	KT935186	scaffold_1: 17203632 - 17204751
	CrGSTF7	KT935187	scaffold_3: 756089 - 756912
	CrGSTF8	KT935188	scaffold_4: 7892156 - 7893036
	CrGSTF9	KT935189	scaffold_4: 7895061 - 7896065
	CrGSTF10	KT935190	scaffold_4: 14896775 - 14897642
	CrGSTF11	KT935191	scaffold_6: 5697480 - 5698285
	CrGSTF12	KT935192	scaffold_6: 15565037 - 15565863

Supplementary Table S1 (continued)

Class	Gene Name	Accession number	Map position (bp)
Theta	*CrGSTT1*	KT935195	scaffold_7: 15658436 - 15659796
Zeta	*CrGSTZ1*	KT935221	scaffold_2: 9214262 - 9216225
	CrGSTZ2	KT935222	scaffold_5: 564319 – 566332
	CrGSTZ3	KT935223	scaffold_5: 566729 - 569295
Lambda	*CrGSTL1*	KT935193	scaffold_5: 10573244 - 10574877
	CrGSTL2	KT935194	scaffold_6: 563382 - 566174
DHAR	*CrDHAR1*	KT935176	scaffold_1: 6785358 - 6786353
	CrDHAR2	KT935177	scaffold_2: 11852999 - 11853919
	CrDHAR3	KT935178	scaffold_6: 5524835 – 5526253
TCHQD	*CrTCHQD1*	KT935224	scaffold_2: 12680754 - 12681659
EF1Bγ	*CrEF1Bγ1*	KT935179	scaffold_1: 3193684 - 3195669
	CrEF1Bγ2	KT935180	scaffold_2: 3094794 - 3096969

Supplementary Table S2 Primers used to amplify *Capsella* GST genes

Genes	Primer name	Sequence(5'-3')
CrGSTU1	CrGSTU1CL1	GGTTCATTCATAATCTCCGGT
	CrGSTU1CL2	ATATTTAAGAGGATCCACATCCTT
CrGSTU2	CrGSTU2CL1	CAAAGCTCTCTGTTTCGGAT
	CrGSTU2CL2	CAAATAAAAAACCTAGTTCACCAG
CrGSTU3	CrGSTU3CL1	AGCTAGATCAAGAGAGCAACCT
	CrGSTU3CL2	CATGGAACAGAACAAAGAAGAG
CrGSTU4	CrGSTU4CL1	AGCTTTAATCAAGATTCAAGAACA
	CrGSTU4CL2	TATAAATATAGCAGTAGAAGTAAGAAGATG
CrGSTU5	CrGSTU5CL1	GAGGTAAACCATACCGGATCT
	CrGSTU5CL2	TTCTTCTATACAACACATGAAAAGAT
CrGSTU6	CrGSTU6CL1	GTTCAGATCAATAAAACCAGGAG
	CrGSTU6CL2	AACTTTGGAAGGAAGCATACAC
CrGSTU7	CrGSTU7CL1	GAACAAGGAGCTTGTAGAACCA
	CrGSTU7CL2	TTGTTTTCACTTTTCAGTGTTTACT
CrGSTU8	CrGSTU8CL1	CAAATCAAGAAAACGTCTATACACA
	CrGSTU8CL2	ACATTGCAATACATGTCTAAGATGA
CrGSTU9	CrGSTU9CL1	TAAATTGAAAATAATTAAATCAAATCA
	CrGSTU9CL2	GCATGCAAATACATTTCTACGA
CrGSTU10	CrGSTU10CL1	GATCAGTCTCGTCAAAAGTGTAAC
	CrGSTU10CL2	GATAGTAATTATTATGAGTCTTTGGTGT
CrGSTU11	CrGSTU11CL1	AGAAATAAAACAACAGAGTTTTGAG
	CrGSTU11CL2	TAACCTGACCAACATCTTGATCT
CrGSTU12	CrGSTU12CL1	CTTCTTCACAGAGTCTCACCGA
	CrGSTU12CL2	GCAAATCAGAAACAGGGAAAT
CrGSTU13	CrGSTU13CL1	CGATAAAGAGTATACATCAAAGAAGAG
	CrGSTU13CL2	GTGAGATGGGGAATCAATAAGA
CrGSTU14	CrGSTU14CL1	AGGAATCGAGAAGAGAAGAGCT
	CrGSTU14CL2	GAAAACGGGAATGGAAGAGAT
CrGSTU15	CrGSTU15CL1	TAATTTGTGAGCGTTTCAGATATC
	CrGSTU15CL2	TGACGAGAAAAGAACAAGAATATG
CrGSTU16	CrGSTU16CL1	GTACAGTAAAGAAGCTCTCTTTTGTA
	CrGSTU16CL2	ACCAAACGCACAAAGAAACA
CrGSTU17	CrGSTU17CL1	GAACCAACTTTTCTTAATACATTATAC
	CrGSTU17CL2	ACAAATATACTAAGAAAGAGGCTCT
CrGSTU18	CrGSTU18CL1	AACTCACAAGAGTGAGTGAGAGAC
	CrGSTU18CL2	CATACTTTGATTATTATGAGAAGTCTCT
CrGSTU19	CrGSTU19CL1	CTAAGAAAGCTAAGGAAGCAGAG
	CrGSTU19CL2	GGATGATCAAGGACATAGAAAGA
CrGSTU20	CrGSTU20CL1	CCACCATTTCTAATTCTAAGACG
	CrGSTU20CL2	ACAGATGATTTGTTTTCTTGAATG

Supplementary Table S2 (continued)

Genes	Primer name	Sequence(5'-3')
CrGSTU21	CrGSTU21CL1	AGAGAGAGAGTTATAGAGAGAAGTGC
	CrGSTU21CL2	TTAATTATACAAACATATGTTGTCTCTT
CrGSTU22	CrGSTU22CL1	AAGTGGGAAAGAAATTAATTGAT
	CrGSTU22CL2	ACAATGCAAATACAAATATGTTGT
CrGSTU23	CrGSTU23CL1	GAAACCAAAAAGTGAACAAGTGA
	CrGSTU23CL2	CAGAACAGAGTTATGATGTTGTCAT
CrGSTU24	CrGSTU24CL1	CAAACAGAGGAGTTGATCCAGT
	CrGSTU24CL2	AAGAAGTACGCAGTTGTGTTTAAT
CrGSTU25	CrGSTU25CL1	CAAAAGAAAAAGTAGAAGGTCAGAC
	CrGSTU25CL2	GCAAACCATTTCCTATAAGCAT
CrGSTF1	CrGSTF1CL1	TATCACTAAAAACAGCATTAACTCAC
	CrGSTF1CL2	CTTTATTCAGTATTGTGACTTAACAGT
CrGSTF2	CrGSTF2CL1	AAAGAAAAACAGCATTTAGTTCAG
	CrGSTF2CL2	TTATTCAATCCTGTGATAGTCAGC
CrGSTF3	CrGSTF3CL1	TTGAATATTCATATATAATTGAGTCGT
	CrGSTF3CL2	ATACACAATGTTCTTTGCTGATCT
CrGSTF4	CrGSTF4CL1	GCACAAATAAGTCTTATCATCATCT
	CrGSTF4CL2	AAGATACAAACACAGTGACACACA
CrGSTF5	CrGSTF5CL1	AGAAAAACAGCATTTCGTTCA
	CrGSTF5CL2	CAATCTTGTGATAGTCAGCTTGAT
CrGSTF6	CrGSTF6CL1	GAGATAACAATCGGACTTGAGATAT
	CrGSTF6CL2	ACTTAACATTGTAGTTTTGATTTTCT
CrGSTF7	CrGSTF7CL1	ACAATTGATATCAATTGTTACGAAT
	CrGSTF7CL2	GTCCCATAAATTAGACAATTAACAT
CrGSTF8	CrGSTF8CL1	GTGAGTAAAACAGAGAAGAAACAAC
	CrGSTF8CL2	CAAACACACAAGAAGAAATCATC
CrGSTF9	CrGSTF9CL1	ATCAAAGAAGTAAGAAAGAAAGAAAG
	CrGSTF9CL2	CAAGTCAACACGAGAACCACA
CrGSTF10	CrGSTF10CL1	CAACAACAAAGTCATCATCTTAGTAT
	CrGSTF10CL2	AAAGAGACCCAAAAGACAAGAG
CrGSTF11	CrGSTF11CL1	GTCATAGCAATAACAAAGTTATATTGT
	CrGSTF11CL2	AACCAGCATATATATTCGAGCTAT
CrGSTF12	CrGSTF12CL1	CTCTACAGAGAGTTTCTAAGTAACCA
	CrGSTF12CL2	TTATAGTGACTGAACCAAAACCAT
CrGSTT1	CrGSTT1CL1	CTGATCCACAAAATCAGAAGAAG
	CrGSTT1CL2	CGGCTTGGATAAGTTTTGTTC
CrGSTZ1	CrGSTZ1CL1	ATGGCTGATATCGGATCCATGGCATACTCCGATGGACT
	CrGSTZ1CL2	GTGGTGGTGGTGCTCGAGTTACTAGTTGGATGGAGCTGGAG

Supplementary Table S2 (continued)

Genes	Primer name	Sequence(5'-3')
CrGSTZ2	CrGSTZ2CL1	ATGGCTGATATCGGATCCATGGCGAATTCTGGCGCAGAT
	CrGSTZ2CL2	GTGGTGGTGGTGCTCGAGTTATCATATGGTGGAAGGAGCAT
CrGSTZ3	CrGSTZ3CL1	ATGGCTGATATCGGATCCATGGCGAATTCTGGCGCAGAG
	CrGSTZ3CL2	GTGGTGGTGGTGCTCGAGTTATCAGATGGTGGAAGGAGCAT
CrGSTL1	CrGSTL1CL1	ATGGCTGATATCGGATCCATGAGTGTCGGAGTTAATGT
	CrGSTL1CL2	GTGGTGGTGGTGCTCGAGTTAAAGTCGTGCTTCTGCTT
CrGSTL2	CrGSTL2CL1	CGAGCTTAGTTTGGTATAATAAAAGT
	CrGSTL2CL2	CCAATGAAATCAATAAAGTATATCCTA
CrDHAR1	CrDHAR1CL1	ATGGCTGATATCGGATCCATGGCTCTCGAAATATGCGT
	CrDHAR1CL2	GTGGTGGTGGTGCTCGAGTTATCAAGGGTTGACCTTAGGAG
CrDHAR2	CrDHAR2CL1	ATGGCTGATATCGGATCCATGGCTCTCGATATCTGCGT
	CrDHAR2CL2	GTGGTGGTGGTGCTCGAGTTATCAAGCGTTCACCTTAGATT
CrDHAR3	CrDHAR3CL1	ATGGCTGATATCGGATCCATGGCGGCGAGTCCTCCTCT
	CrDHAR3CL2	GTGGTGGTGGTGCTCGAGTTAACCCATCACCTTTGGTC
CrTCHQD1	CrTCHQD1CL1	ATGGCTGATATCGGATCCATGCAGTTATATCATCATCC
	CrTCHQD1CL2	GTGGTGGTGGTGCTCGAGTTATCAATATTTCCTGAGCAAAC
CrEF1Bγ1	CrEF1Bγ1CL1	ATGGCTGATATCGGATCCATGGCTTTGGTCTTGCACACG
	CrEF1Bγ1CL2	GTGGTGGTGGTGCTCGAGTCACTTGAAGCACTTGGCGT
CrEF1Bγ2	CrEF1Bγ2CL1	ATGGCTGATATCGGATCCATGGCTTTGGTCTTGCACAC
	CrEF1Bγ2CL2	GTGGTGGTGGTGCTCGAGTTATCACTTAAAGCATTTGGCGT

Supplementary Table S3 PCR primers used to detect the expression of *Capsella* GST genes

**Genes**	**Primer name**	**Sequence(5'-3')**
CrGSTU1	CrGSTU1RT1	GGTTCATTCATAATCTCCGGT
	CrGSTU1RT2	ATATTTAAGAGGATCCACATCCTT
CrGSTU2	CrGSTU2RT1	CAAAGCTCTCTGTTTCGGAT
	CrGSTU2RT2	CAAATAAAAAACCTAGTTCACCAG
CrGSTU3	CrGSTU3RT1	AGCTAGATCAAGAGAGCAACCT
	CrGSTU3RT2	CATGGAACAGAACAAAGAAGAG
CrGSTU4	CrGSTU4RT1	AGCTTTAATCAAGATTCAAGAACA
	CrGSTU4RT2	TATAAATATAGCAGTAGAAGTAAGAAGATG
CrGSTU5	CrGSTU5RT1	GAGGTAAACCATACCGGATCT
	CrGSTU5RT2	TTCTTCTATACAACACATGAAAAGAT
CrGSTU6	CrGSTU6RT1	GTTCAGATCAATAAAACCAGGAG
	CrGSTU6RT2	AACTTTGGAAGGAAGCATACAC
CrGSTU7	CrGSTU7RT1	GAACAAGGAGCTTGTAGAACCA
	CrGSTU7RT2	TTGTTTTCACTTTTCAGTGTTTACT
CrGSTU8	CrGSTU8RT1	CAAATCAAGAAAACGTCTATACACA
	CrGSTU8RT2	ACATTGCAATACATGTCTAAGATGA
CrGSTU9	CrGSTU9RT1	TAAATTGAAAATAATTAAATCAAATCA
	CrGSTU9RT2	GCATGCAAATACATTTCTACGA
CrGSTU10	CrGSTU10RT1	GATCAGTCTCGTCAAAAGTGTAAC
	CrGSTU10RT2	GATAGTAATTATTATGAGTCTTTGGTGT
CrGSTU11	CrGSTU11RT1	AGAAATAAAACAACAGAGTTTTGAG
	CrGSTU11RT2	TAACCTGACCAACATCTTGATCT
CrGSTU12	CrGSTU12RT1	CTTCTTCACAGAGTCTCACCGA
	CrGSTU12RT2	GCAAATCAGAAACAGGGAAAT
CrGSTU13	CrGSTU13RT1	CGATAAAGAGTATACATCAAAGAAGAG
	CrGSTU13RT2	GTGAGATGGGGAATCAATAAGA
CrGSTU14	CrGSTU14RT1	AGGAATCGAGAAGAGAAGAGCT
	CrGSTU14RT2	GAAAACGGGAATGGAAGAGAT
CrGSTU15	CrGSTU15RT1	TAATTTGTGAGCGTTTCAGATATC
	CrGSTU15RT2	TGACGAGAAAAGAACAAGAATATG
CrGSTU16	CrGSTU16RT1	GTACAGTAAAGAAGCTCTCTTTTGTA
	CrGSTU16RT2	ACCAAACGCACAAAGAAACA
CrGSTU17	CrGSTU17RT1	GAACCAACTTTTCTTAATACATTATAC
	CrGSTU17RT2	ACAAATATACTAAGAAAGAGGCTCT
CrGSTU18	CrGSTU18RT1	AACTCACAAGAGTGAGTGAGAGAC
	CrGSTU18RT2	CATACTTTGATTATTATGAGAAGTCTCT
CrGSTU19	CrGSTU19RT1	CTAAGAAAGCTAAGGAAGCAGAG
	CrGSTU19RT2	GGATGATCAAGGACATAGAAAGA

Supplementary Table S3 (continued)

Genes	Primer name	Sequence(5’-3’)
CrGSTU20	CrGSTU20RT1	CCACCATTTCTAATTCTAAGACG
	CrGSTU20RT2	ACAGATGATTTGTTTTCTTGAATG
CrGSTU21	CrGSTU21RT1	AGAGAGAGAGTTATAGAGAGAAGTGC
	CrGSTU21RT2	TTAATTATACAAACATATGTTGTCTCTT
CrGSTU22	CrGSTU22RT1	AAGTGGGAAAGAAATTAATTGAT
	CrGSTU22RT2	ACAATGCAAATACAAATATGTTGT
CrGSTU23	CrGSTU23RT1	GAAACCAAAAAGTGAACAAGTGA
	CrGSTU23RT2	CAGAACAGAGTTATGATGTTGTCAT
CrGSTU24	CrGSTU24RT1	CAAACAGAGGAGTTGATCCAGT
	CrGSTU24RT2	AAGAAGTACGCAGTTGTGTTTAAT
CrGSTU25	CrGSTU25RT1	CAAAAGAAAAAGTAGAAGGTCAGAC
	CrGSTU25RT2	GCAAACCATTTCCTATAAGCAT
CrGSTF1	CrGSTF1RT1	TATCACTAAAAACAGCATTAACTCAC
	CrGSTF1RT2	CTTTATTCAGTATTGTGACTTAACAGT
CrGSTF2	CrGSTF2RT1	AAAGAAAAACAGCATTTAGTTCAG
	CrGSTF2RT2	TTATTCAATCCTGTGATAGTCAGC
CrGSTF3	CrGSTF3RT1	TTGAATATTCATATATAATTGAGTCGT
	CrGSTF3RT2	ATACACAATGTTCTTTGCTGATCT
CrGSTF4	CrGSTF4RT1	GCACAAATAAGTCTTATCATCATCT
	CrGSTF4RT2	AAGATACAAACACAGTGACACACA
CrGSTF5	CrGSTF5RT1	AGAAAAACAGCATTTCGTTCA
	CrGSTF5RT2	CAATCTTGTGATAGTCAGCTTGAT
CrGSTF6	CrGSTF6RT1	GAGATAACAATCGGACTTGAGATAT
	CrGSTF6RT2	ACTTAACATTGTAGTTTTGATTTTCT
CrGSTF7	CrGSTF7RT1	ACAATTGATATCAATTGTTACGAAT
	CrGSTF7RT2	GTCCCATAAATTAGACAATTAACAT
CrGSTF8	CrGSTF8RT1	GTGAGTAAAACAGAGAAGAAACAAC
	CrGSTF8RT2	CAAACACACAAGAAGAAATCATC
CrGSTF9	CrGSTF9RT1	ATCAAAGAAGTAAGAAAGAAAGAAAG
	CrGSTF9RT2	CAAGTCAACACGAGAACCACA
CrGSTF10	CrGSTF10RT1	CAACAACAAAGTCATCATCTTAGTAT
	CrGSTF10RT2	AAAGAGACCCAAAAGACAAGAG
CrGSTF11	CrGSTF11RT1	GTCATAGCAATAACAAAGTTATATTGT
	CrGSTF11RT2	AACCAGCATATATATTCGAGCTAT
CrGSTF12	CrGSTF12RT1	CTCTACAGAGAGTTTCTAAGTAACCA
	CrGSTF12RT2	TTATAGTGACTGAACCAAAACCAT
CrGSTT1	CrGSTT1RT1	CTGATCCACAAAATCAGAAGAAG
	CrGSTT1RT2	CGGCTTGGATAAGTTTTGTTC

Supplementary Table S3 (continued)

Genes	Primer name	Sequence(5'-3')
CrGSTZ1	CrGSTZ1RT1	ATGGCTGATATCGGATCCATGGCATACTCCGATGGACT
	CrGSTZ1RT2	GTGGTGGTGGTGCTCGAGTTACTAGTTGGATGGAGCTGGAG
CrGSTZ2	CrGSTZ2RT1	ATGGCTGATATCGGATCCATGGCGAATTCTGGCGCAGAT
	CrGSTZ2RT2	GTGGTGGTGGTGCTCGAGTTATCATATGGTGGAAGGAGCAT
CrGSTZ3	CrGSTZ3RT1	ATGGCTGATATCGGATCCATGGCGAATTCTGGCGCAGAG
	CrGSTZ3RT2	GTGGTGGTGGTGCTCGAGTTATCAGATGGTGGAAGGAGCAT
CrGSTL1	CrGSTL1RT1	ATGGCTGATATCGGATCCATGAGTGTCGGAGTTAATGT
	CrGSTL1RT2	GTGGTGGTGGTGCTCGAGTTAAAGTCGTGCTTCTGCTT
CrGSTL2	CrGSTL2RT1	CGAGCTTAGTTTGGTATAATAAAAGT
	CrGSTL2RT2	CCAATGAAATCAATAAAGTATATCCTA
CrDHAR1	CrDHAR1RT1	ATGGCTGATATCGGATCCATGGCTCTCGAAATATGCGT
	CrDHAR1RT2	GTGGTGGTGGTGCTCGAGTTATCAAGGGTTGACCTTAGGAG
CrDHAR2	CrDHAR2RT1	ATGGCTGATATCGGATCCATGGCTCTCGATATCTGCGT
	CrDHAR2RT2	GTGGTGGTGGTGCTCGAGTTATCAAGCGTTCACCTTAGATT
CrDHAR3	CrDHAR3RT1	ATGGCTGATATCGGATCCATGGCGGCGAGTCCTCCTCT
	CrDHAR3RT2	GTGGTGGTGGTGCTCGAGTTAACCCATCACCTTTGGTC
CrTCHQD1	CrTCHQD1RT1	ATGGCTGATATCGGATCCATGCAGTTATATCATCATCC
	CrTCHQD1RT2	GTGGTGGTGGTGCTCGAGTTATCAATATTTCCTGAGCAAAC
CrEF1Bγ1	CrEF1Bγ1RT1	ATGGCTGATATCGGATCCATGGCTTTGGTCTTGCACACG
	CrEF1Bγ1RT2	GTGGTGGTGGTGCTCGAGTCACTTGAAGCACTTGGCGT
CrEF1Bγ2	CrEF1Bγ2RT1	ATGGCTGATATCGGATCCATGGCTTTGGTCTTGCACAC
	CrEF1Bγ2RT2	GTGGTGGTGGTGCTCGAGTTATCACTTAAAGCATTTGGCGT
CrAction	CrActionRT1	ATCCAAGCTGTTCTATCCTTGTAT
	CrActionRT2	TTGGTGCAAGTGCTGTGATT

Supplementary Table S4 Primers used to construct the protein expression vector

Genes	Primer name	Sequence(5’-3’)
CrGSTU1	CrGSTU1EX1	AGATATCATGGCGGCCGATGAAGTGA
	CrGSTU1EX2	TAAGCTTTTATCAGGCTTGCGGCTTAGGA
CrGSTU2	CrGSTU2EX1	AGGATCCATGGCAAGCGGCGAAGTGAAG
	CrGSTU2EX2	TCTCGAGTTAGGCCTGCGGCTTGGCAAAG
CrGSTU3	CrGSTU3EX1	AGATATCATGACGGAGGAGGTGATTCTTCTG
	CrGSTU3EX2	TAAGCTTTTACTCTAGTTCAAGTTTCTTCCTACGCT
CrGSTU4	CrGSTU4EX1	AGATATCATGGCGGACGAGGTGATTCTT
	CrGSTU4EX2	TAAGCTTTTACTATTCGATCCCAATATGTTTCTTTAGC
CrGSTU5	CrGSTU5EX1	AGATATCATGGCGGACGAAGTGATTCTTCTT
	CrGSTU5EX2	TAAGCTTTTATCATGCTCCAAATTTCTTCCTCAAG
CrGSTU6	CrGSTU6EX1	AGGATCCATGGCTCAGAATGATACAGTGAC
	CrGSTU6EX2	TCTCGAGTTATCACTGAACACTGAACTTCTTCTTTG
CrGSTU7	CrGSTU7EX1	AGGATCCATGGGGAAAGAGAATGGCAAC
	CrGSTU7EX2	TGTCGACTTATCATTCAACACCAAAAATCTGTCT
CrGSTU8	CrGSTU8EX1	AGATATCATGGGAGAAATAATGGAAGTGAAACTATT
	CrGSTU8EX2	TGTCGACTTACTAAGATCTTGAAGCTGCGGA
CrGSTU10	CrGSTU10EX1	AGGATCCATGGGTCTAATGAATGGTTCG
	CrGSTU10EX2	TCTCGAGTTATCATTTAAAGATTGAAGTATTGAACTTGA
CrGSTU11	CrGSTU11EX1	AGATATCATGGAGGACCAGAGCAACAAAGTGAC
	CrGSTU11EX2	TAAGCTTTTACGCACTTGCAGCCTGCTG
CrGSTU12	CrGSTU12EX1	AGATATCATGGCAGAAGAGGTTATTCTACTGG
	CrGSTU12EX2	TAAGCTTTTATCAGAGTCCTAAAACAGTCTTCCG
CrGSTU13	CrGSTU13EX1	AGGATCCATGGCGGATGAAGTGATCCT
	CrGSTU13EX2	TCTCGAGTTAGACACCATATTCCTTCCTAAGCTTATAG
CrGSTU14	CrGSTU14EX1	AGGATCCATGGGAGACGAAGTGATCCT
	CrGSTU14EX2	TCTCGAGTTACTGGACTCCATAAACCTTCTTAAG
CrGSTU15	CrGSTU15EX1	AGGATCCATGGCGAACAAACCGATTCT
	CrGSTU15EX2	TCTCGAGTTACTACTCAGATCCGAGATTGTTCTTC
CrGSTU16	CrGSTU16EX1	AGGATCCATGACGAACGAGGTGATCCT
	CrGSTU16EX2	TCTCGAGTTACTACTCAGCTACAAATTTCGTCCT
CrGSTU17	CrGSTU17EX1	AGGATCCATGAATCAAGAAGAGCAAGTACAGGT
	CrGSTU17EX2	TCTCGAGTTAACCAGATGTAGCAGCACTTCTAAAC
CrGSTU18	CrGSTU18EX1	AGGATCCATGGCTGAGAGATCAGAAGATTC
	CrGSTU18EX2	TCTCGAGTTATCAAGCTGATTTGATATTAAGCTTGTC
CrGSTU19	CrGSTU19EX1	AGGATCCATGCCCGAGAATGAAGAAGT
	CrGSTU19EX2	TCTCGAGTTAAGAAGATCTTACTCTCTGTGT
CrGSTU20	CrGSTU20EX1	AGGATCCATGGCGGAGAATGAAGAAGT
	CrGSTU20EX2	TCTCGAGTTAAGAAGAACTTACTCTCTCAACCTTAT

Supplementary Table S4 (continued)

Genes	Primer name	Sequence(5'-3')
CrGSTU21	CrGSTU21EX1	AGATATCATGGCAGAGAAAGGTGAGGATGTG
	CrGSTU21EX2	TGTCGACTTAAGCTGATTTGATTCGTTCTGC
CrGSTU22	CrGSTU22EX1	AGATATCATGGCTGAGAAAGAAGAGGGTGTGA
	CrGSTU22EX2	TGTCGACTTACTAGGTTGCTTTCATTAGCTTTACAAT
CrGSTU23	CrGSTU23EX1	AGGATCCATGGAGAAGAAAGAAGAGAGTGT
	CrGSTU23EX2	TCTCGAGTTAGGAAGACTTAACTCTCTCAATAATT
CrGSTU24	CrGSTU24EX1	AGGATCCATGGCGGAGAAAGAAGAGAGT
	CrGSTU24EX2	TCTCGAGTTAGGCAGACATAATTTTCTCTGCAAT
CrGSTU25	CrGSTU25EX1	AGGATCCATGTCAGAAGAGGAAGTGGTG
	CrGSTU25EX2	TCTCGAGTTATCAATAACCATAGAAGAACTCGTG
CrGSTF1	CrGSTF1EX1	AGGATCCATGGCAGGAATCAAAGTTTTC
	CrGSTF1EX2	TCTCGAGTTAGAGAACCTTCTGAGCAGAAGG
CrGSTF2	CrGSTF2EX1	AGGATCCATGGCAGGAATCAAAGTTTTC
	CrGSTF2EX2	TCTCGAGTTAAAGAACCTTCTGAGCAGAAGG
CrGSTF4	CrGSTF4EX1	AGATATCATGGAGTGCTTACAGATGGTGTTCA
	CrGSTF4EX2	TGTCGACTTACTTCTGCTTTGATTTAGTTCTTAGCTT
CrGSTF5	CrGSTF5EX1	AGGATCCATGGCAGGAATCCAAGTTTT
	CrGSTF5EX2	TCTCGAGTTAAAGAACCTTCTGAGCAGAAGG
CrGSTF6	CrGSTF6EX1	AGGATCCATGGCTGATTCGAAGATGAAG
	CrGSTF6EX2	TCTCGAGTTAGTTGTTCCCCATCGCAAAC
	CrGSTF6PJ1	CTCCATCTTCAAGAACCGGGACTTCACCAAATGGGTTGAGAGTAGAGAGAAA
	CrGSTF6PJ2	CCGGTTCTTGAAGATGGAGATCTCAAACTTTACGAATCAAAGGCGATCACGC
CrGSTF7	CrGSTF7EX1	AGATATCATGGTGGTCAAAGTGTATGGGCAGATA
	CrGSTF7EX2	TGTCGACTTAGTAGGTAGCCAATTCCATGAGCTT
CrGSTF8	CrGSTF8EX1	AGGATCCATGGTGCTAAAGGTGTACGGA
	CrGSTF8EX2	TCTCGAGTTAACCCGGGAGTGAAAACTT
CrGSTF9	CrGSTF9EX1	AGGATCCATGGTGTTGAAGATCTATGCTCC
	CrGSTF9EX2	TCTCGAGTTAAACAGGTAACGCGTACTTCTCA
CrGSTF10	CrGSTF10EX1	AGGATCCATGGCAAGTATCAAGGTTCACG
	CrGSTF10EX2	TCTCGAGTTATCACAGCTTCTGGAGGTCAAT
CrGSTF11	CrGSTF11EX1	AGGATCCATGGTTGTGAAACTATACGGGCAG
	CrGSTF11EX2	TCTCGAGTTATCAGTGACGACCAGCCAACTC
CrGSTF12	CrGSTF12EX1	AGGATCCATGGCAGGTATCAAAGTTTTCG
	CrGSTF12EX2	TCTCGAGTTACTGAAGGATCTTCTGGGAAGCT
CrGSTZ1	CrGSTZ1EX1	ATGGCTGATATCGGATCCATGGCATACTCCGATGGACT
	CrGSTZ1EX2	GTGGTGGTGGTGCTCGAGTTACTAGTTGGATGGAGCTGGAG
CrGSTZ2	CrGSTZ2EX1	ATGGCTGATATCGGATCCATGGCGAATTCTGGCGCAGAT
	CrGSTZ2EX2	GTGGTGGTGGTGCTCGAGTTATCATATGGTGGAAGGAGCAT

Supplementary Table S4 (continued)

Genes	Primer name	Sequence(5’-3’)
CrGSTZ3	CrGSTZ3EX1	ATGGCTGATATCGGATCCATGGCGAATTCTGGCGCAGAG
	CrGSTZ3EX2	GTGGTGGTGGTGCTCGAGTTATCAGATGGTGGAAGGAGCAT
CrDHAR1	CrDHAR1EX1	ATGGCTGATATCGGATCCATGGCTCTCGAAATATGCGT
	CrDHAR1EX2	GTGGTGGTGGTGCTCGAGTTATCAAGGGTTGACCTTAGGAG
CrDHAR2	CrDHAR2EX1	ATGGCTGATATCGGATCCATGGCTCTCGATATCTGCGT
	CrDHAR2EX2	GTGGTGGTGGTGCTCGAGTTATCAAGCGTTCACCTTAGATT
CrDHAR3	CrDHAR3EX1	ATGGCTGATATCGGATCCATGGCGGCGAGTCCTCCTCT
	CrDHAR3EX2	GTGGTGGTGGTGCTCGAGTTAACCCATCACCTTTGGTC
	CrEF1Bγ2EX2	GTGGTGGTGGTGCTCGAGTTATCACTTAAAGCATTTGGCGT
